# Introduction of a Methodology to Enhance the Stabilization Process of PAN Fibers by Modeling and Advanced Characterization

**DOI:** 10.3390/ma13122749

**Published:** 2020-06-17

**Authors:** George Konstantopoulos, Spyros Soulis, Dimitrios Dragatogiannis, Costas Charitidis

**Affiliations:** Research Unit of Advanced, Composite, Nano Materials & Nanotechnology, School of Chemical Engineering, National Technical University of Athens, Department III, 9 Heroon Polytechniou str., Zografou Campus, 157 73 Athens, Greece; gkonstanto@chemeng.ntua.gr (G.K.); sksoulis@gmail.com (S.S.); ddragato@chemeng.ntua.gr (D.D.)

**Keywords:** polyacrylonitrile, stabilization, cyclization, kinetics, carbon fiber

## Abstract

A methodology for designing the oxidative stabilization process of polyacrylonitrile (PAN) fibers is examined. In its core, this methodology is based on a model that describes the characteristic fiber length variation during thermal processing, through the de-convolution of three main contributors (i.e., entropic and chemical shrinkage and creep elongation). The model demonstrated an additional advantage of offering further insight into the physical and chemical phenomena taking place during the treatment. Validation of PAN-model prediction performance for different processing parameters was achieved as demonstrated by Fourier Transform Infrared Spectroscopy (FTIR) and Differential Scanning Calorimetry (DSC). Τensile testing revealed the effect of processing parameters on fiber quality, while model prediction demonstrated that ladder polymer formation is accelerated at temperatures over 200 °C. Additionally, according the DSC and FTIR measurements predictions from the application of the model during stabilization seem to be more precise at high-temperature stabilization stages. It was shown that mechanical properties could be enhanced preferably by including a treatment step below 200 °C, before the initiation of cyclization reactions. Further confirmation was provided via Raman spectroscopy, which demonstrated that graphitic like planes are formed upon stabilization above 200 °C, and thus multistage stabilization is required to optimize synthesis of carbon fibers. Optical Microscopy proved that isothermal stabilization treatment did not severely alter the cross section geometry of PAN fiber monofilaments.

## 1. Introduction

The constantly growing demand in carbon fiber reinforced plastics (CFRPs) in performance-demanding fields such as (aero-)space engineering, automotive, construction, and sport equipment highlights the relative importance of further improving and optimizing the production process and surface chemistry of carbon fibers (CF) in order to fabricate cost- and energy-efficient composites [1,2,3]. This can be better achieved by modifications of the stabilization process [4,5,6,7,8,9,10], since carbonization is a straightforward process that cannot be essentially altered and that investigation on novel CF precursors is time a consuming procedure. Polyacrylonitrile (PAN)-based CFs are more efficiently manufactured (due to their high carbon yield) and demonstrate a structure with less defects and voids, affording CF with high tensile strength in comparison to CFs derived from pitch, Rayon, or renewable precursors [1,10,11,12,13,14,15]. Given that PAN-based CFs represent about 80–90% of the world production, there is a strong incentive for improving the process design, so that it might be further optimized [9,14,16].

Stabilization is considered as the most crucial step and obtaining a proper structure is critical for achieving high performance CFs. This oxidation step, leads to an infusible and inflammable ladder polymer structure, suitable for carbonization [11,14,17,18,19,20]. Regarding the thermal treatment, processability of PAN fibers is enhanced at temperatures exceeding glass transition temperature (T_g_), commonly above 170 °C [21]. In this temperature region the PAN precursor is partially plasticized and this is the point where an improvement in fiber orientation could be achieved, while tension assists the process and leads to enhanced molecular alignment in fibers [22,23,24]. Molecular structure is affected by several reaction pathways that transform the polymer backbone of fused heteroaromatic structures; furthermore, the presence of heteroatoms such as residual hydrogen, nitrogen and oxygen can lead to crosslinking, and thus to higher carbon yields and an improved structure upon carbonization [6,11,25,26,27,28,29].

During the stabilization process, physical and chemical transformations occur that can be observed by the change in fiber length. The latter is dominated by three phenomena, namely: entropic shrinkage, creep, and chemical shrinkage [22,30,31]. The utilization of length change for the in-depth investigation of the stabilization process makes this approach based on the deconvolution of the corresponding phenomena that take place during the process. Entropic shrinkage (ES) or recovery is a physical transformation that occurs at early stabilization stages upon initiation of stabilization reactions. It is attributed to relaxation of highly orientated PAN macromolecules, due to the spinning and post-spinning treatment and the reduction of crystalline regions during thermal treatment. It was found that maximum Entropic Shrinkage (ES_max_, i.e., the reduction of the PAN fibers length during the primal stage of oxidative stabilization in the limiting case where no stress is applied), is a physical constant that depends on the structure of the PAN homo/co-polymer fiber [1]. This phenomenon is derived by the helical form of PAN macromolecule and the interaction in an intermolecular level, which is attributed to repulsive forces of the adjacent carbon-nitrile tribble bond dipoles [24]. Another origin connected to fiber shrinkage is the relaxation effect induced by the heat flux during thermal processing; the pre-existing stretching-induced strains amongst macromolecules by the fiber manufacturing are rearranged to a random and lower energy configuration [1,22,32,33]. In contrast to entropic shrinkage, creep is a heat activated phenomenon which is dependent on temperature and applied stress. These parameters can be adjusted in order to prevent shrinkage, retain post-spinning treatment orientation, and further orientate PAN molecules [22,23,28,32,34]. However, fiber stretching during oxidative stabilization should be applied sparingly, as it is possible to disturb the preferred orientation and hinder the cyclization propagation due to extensive debonding, and thus lead to reduction of mechanical properties [1,13,24,35]. Chemically-induced shrinkage is derived by exothermic chemical reactions, which lead to ladder polymer structure, and it was shown that chemically-induced shrinkage could be used to monitor the nitrile cyclization yield [1,24,33]. The kinetics and extend of length change depends on factors such as the atmosphere, temperature, the applied stress, stretch given to precursor fiber during spinning process, as well as the heating rate [1,13,24,36].

**Figure 1:**
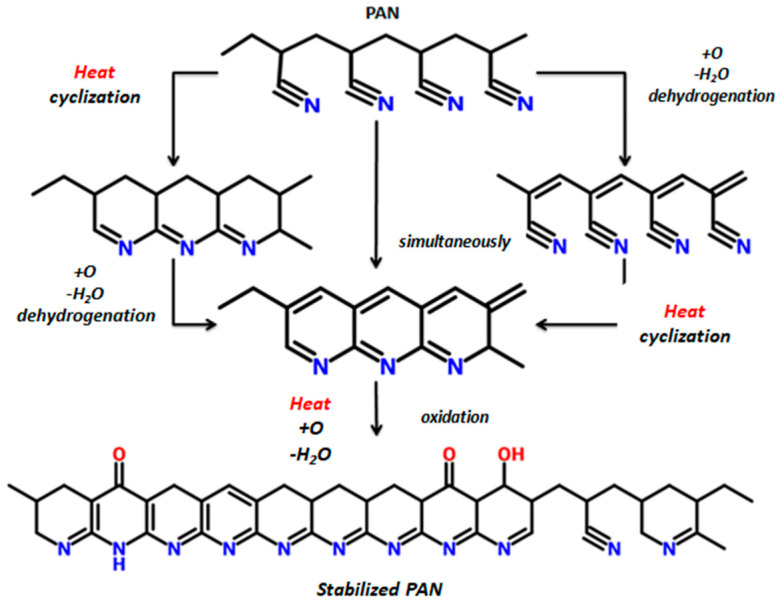
Stabilization mechanism of polyacrylonitrile (PAN) precursor copolymer.

Generally, the main chemical reactions that occur during stabilization are dehydrogenation, cyclization, and oxidation. Their initiation depends on the treatment temperature, which also determines the relative effect and the relative rate of each reaction at the specific treatment stage [7,11,13,14,24,29,32,37]. Dehydrogenation reactions are considered to precede cyclization, while the oxidation takes place in the whole temperature region of stabilization. These reactions are responsible for the gradual colour change of the precursor fiber, starting from white to yellow, to brown, and further spectrum to obtain black colour by the end of dehydrogenation [24,38]. The colour change is assumed to be result of polyene structures produced during stabilization of PAN, while it is also claimed that black colour is attributed to the absorption of the visible spectrum wavelengths by the condensed ring structure. This structure is formed by cyclization-induced carbon-nitrogen double bonds (Figure 1), known as ladder structure. Heating of PAN fibers in an air atmosphere in the low stabilization temperature ranges initiates dehydrogenation reaction, and double bonds are introduced in the chain backbone, which improve thermal stability of the chain [7,29].

Cyclization is considered the dominant stabilization reaction, which is the main occurring reaction when temperature is raised in the region from 180 to 250 °C. The end products are various imine structures among PAN macromolecules though oligomerization of nitrile groups, via linkage between nitrogen and the carbon atom of the succeeding nitrile group of the chain [7,11]. Cyclization is initiated at several activation spots of PAN macromolecule during thermal processing, and evolves intil its growth is terminated by reaching another conjugated or cyclized unit [24]. Numerous reaction paths have been introduced in the literature, based on either self-initiation (mainly in the case of copolymers with oxygen-containing comonomers that act as initiation points) or on external initiation (from the attack of the oxygen on the polymer backbone) [24,39]. Initiation occurs via a radical polymerization mechanism in regards to the homopolymer PAN and through an ionic mechanism, due to the presence of polar functional groups in PAN copolymers, which acts as electron donners, however, the reaction mechanism (or even the sequence of the reactions) does not really affect the structure of stabilized PAN fibers [6,13,24]. However, it is widely accepted that chemical shrinkage, is induced exclusively by the nitrile cyclization reactions [19,40,41].

**Figure 2:**
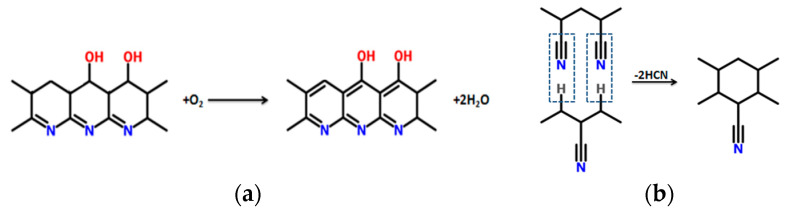
Reactions in presence of oxygen at 300–400 °C of (**a**) aromatization and (**b**) intermolecular cross-linking.

**Figure 3:**
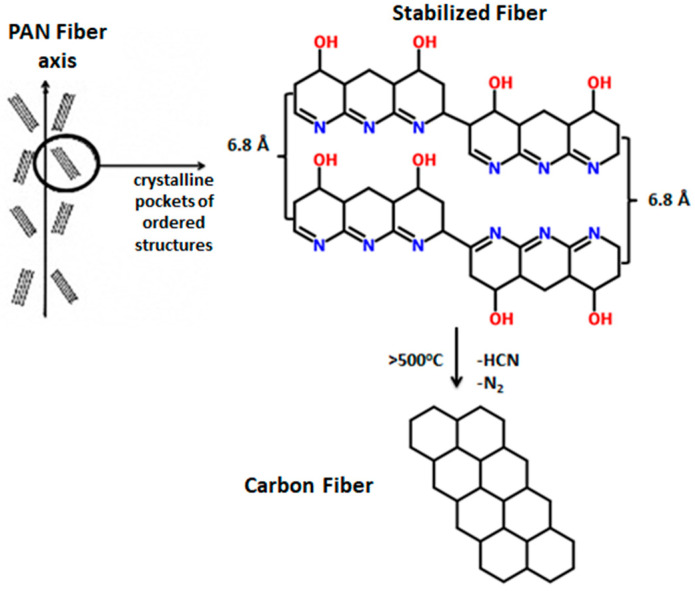
Carbonization mechanism of PAN fibers.

Cyclization is followed by higher temperature oxidation, in order to derive the final pyridine structure. Generally, oxidation has a twofold effect on the stabilization process: oxygen can initiate the formation of active cyclization centers above 240 °C as oxygen diffusion is the dominating pathway for the transformation of the PAN fibers [13,24]. It has also been stated that at elevated temperatures, oxidation reaction occur at a higher rate than cyclization, even though between 180 °C and 240 °C cyclization is the dominating reaction [1,7]. However, it has been reported that further aromatization and intermolecular crosslinking reactions occur in the fiber while performing oxidative stabilization at 300–400 °C (Figure 2). This leads to formation of highly aligned and compact structural units in the stabilized fiber (Figure 3) [24,25]. Molecular chains in the fiber undergo stereochemical transformations, and reinforcement, to form pockets of small-sized basic structural units that maintain a crystalline distance of 6.8 Å [24]. As the stabilization temperature increases, the polymerization kinetics are accelerated.

In most case studies, the object of stabilization investigation is the description of reaction mechanisms and the effect on structural changes; using microscopy, spectroscopy, and thermal analysis kinetic studies on cyclization and oxidation are performed, and progress of the reactions (e.g., cyclization) is estimated [19,30,31,40,42]. While this approach seems time consuming, industrial scale stabilization can be better optimized through a combination of time-consuming experimentation, together with the application of empirical laws that relate temperature, tension, and time with the mechanical performance of the synthesized CFs [29].

The aim of this investigation is to develop a descriptive model that will correlate the length change during stabilization of PAN fibers in the variation range of stabilization main parameters (temperature, time, and applied stress) and correlate it with the structural changes. Such a model could use the length change during the process as an effective control parameter. The foundation of the model is to monitor the main stabilization reactions by using macroscopic observations on the change in fiber length, as well as to establish the relation between length variation and stabilization parameters for each one of these phenomena. Since the correlation between cyclization and chemical shrinkage has been well established, the latter can be used as a measure to quantify the estimation of the stabilization progress, given that cyclization is considered as the most crucial reaction during the formation of the ladder polymer. By using the extracted model, it is expected that the number of the necessary experiments for optimizing the process will be minimized. Fourier Transform Infrared Spectroscopy (FTIR) was used to monitor the bonds formation and Differential Scanning Calorimetry (DSC) to measure the energy evolution during thermal processing, in order to quantify cyclization yield and compare the results to the model predictions. Further characterization was also performed to identify structural transformations during stabilization, such as optical microscopy in order to measure the deviation from circularity of fiber cross section.

## 2. Materials and Methods

### 2.1. Materials

Polyacrylonitrile (PAN) copolymer fiber yarns were obtained by Shenzhen Yataida High-Tech Co., Ltd. (YTD PAN fibers), Shenzhen, China, consisting of 1000 filaments (fineness cv% ≤ 13%). The YTD PAN fibers and were not subjected to any treatment prior to stabilization. 

### 2.2. Isothermal Stabilization Process

Stabilized fibers were synthesized in a stabilization furnace apparatus with internal air circulation, temperature control, and load adjustment capability. In order to be able to measure the length variation during the treatment, the fibers were inserted in the oven using a proper frame. To enhance reproducibility, the samples that were prepared for stabilization had the same initial fiber length and were hanged on the frame on pre-specified positions (i.e., their position inside the stabilization oven is well defined). Specimens were heated isothermally in the temperature range between 160–270 °C, with the applied tension ranging between 0.07 and 22 MPa for different process time, which could be extended depending on the needs of modeling stabilization kinetics for the copolymer fiber.

### 2.3. Characterization of Stabilized YTD PAN Fibers

Fourier Transform Infrared Reflectance (FTIR) was performed on samples prepared using KBr pellet technique on an Agilent Cary 630 spectrometer (Agilent, Santa Clara, CA, USA). The operating wavelength was set in the range of 4000–400 cm^−1^ with resolution of 4 cm^−1^. Samples were dried overnight at 60 °C, and stabilized PAN fibers were grinded with KBr powder at 2% wt. mixing ratio to comply with the quantification protocol similarly to [43]. Then, the powder was pressed into pellets and measured using the proper FTIR transmission module. Raman spectroscopy was performed using InviaH-Renishaw argon laser with wavelength 514.5 nm (Green) and laser beam size of ~2 µm of diameter. A 50× magnification microscope and 10 s exposure with 5 accumulations for each scan were used. Scanning spectrum from 100 to 3500 cm^−1^ was examined to obtain both the first- and second-order Raman bands of carbon materials. Laser power was set at 0.3 mW, 5% of the maximum available power of 6 mW. The correction with respect to the quantum efficiency of detector and the baseline correction have been applied using MATLAB software (MATLAB and Statistics Toolbox Release 2012b, The MathWorks, Inc., Natick, MA, USA). In order to make the comparisons, all the spectra normalized to the D peak at 1372 cm^−1^.

For thermal analysis, a simultaneous TG-DTA/DSC measurement was performed using the NETZSCH STA 449 F5 Jupiter Apparatus (NETZSCH-Gerätebau GmbH, Selb, Germany). The system consists of a Silicon Carbide Furnace with temperature range from 25 °C to 1550 °C, SiC heating element and Al_2_O_3_ protective tube for gas flow with stop valve. Thermogravimetric Analysis (TGA) experiments were conducted using N_2_ or synthetic air flow (mix of 80% N_2_ and 20% O_2_) of 50 mL/min, and a heating rate of 5 °C/min. Characterized PAN samples were oxidatively stabilized in a thermal furnace apparatus under 300 g of applied load for 4 h, in the temperature range of 160 to 270 °C (isothermally), prior to characterization.

In order to investigate any deviation in cross-section circularity of PAN fibers induced by stabilization, OM was performed using a Axio Imager A2m optical microscope and AxioCam ICc5 CCD camera (Carl Zeiss, Oberkochen, Germany), while Image analysis software was used named ImageJ (ImageJ 1.53b, U. S. National Institutes of Health, Maryland, USA). For OM investigation, four monofilaments were selected from each strand with random sampling. The aim of this investigation was to study the variation in PAN fiber cross-section after the stabilization treatment, considering that fiber circularity may be affected by extreme temperature and stress conditions. Prior to their measurement, the samples were encapsulated using a commercial polymer in powder form and a suitable organic solvent was used at a 1:1 ratio to produce a liquefied resin. The resin was hardened for 24 h followed by grinding progressively with decreasing granulometry reaching a final grain size of 10 μm.

## 3. Results and Discussion

### 3.1. Determination of Entropic Shrinkage (ES)

**Figure 4:**
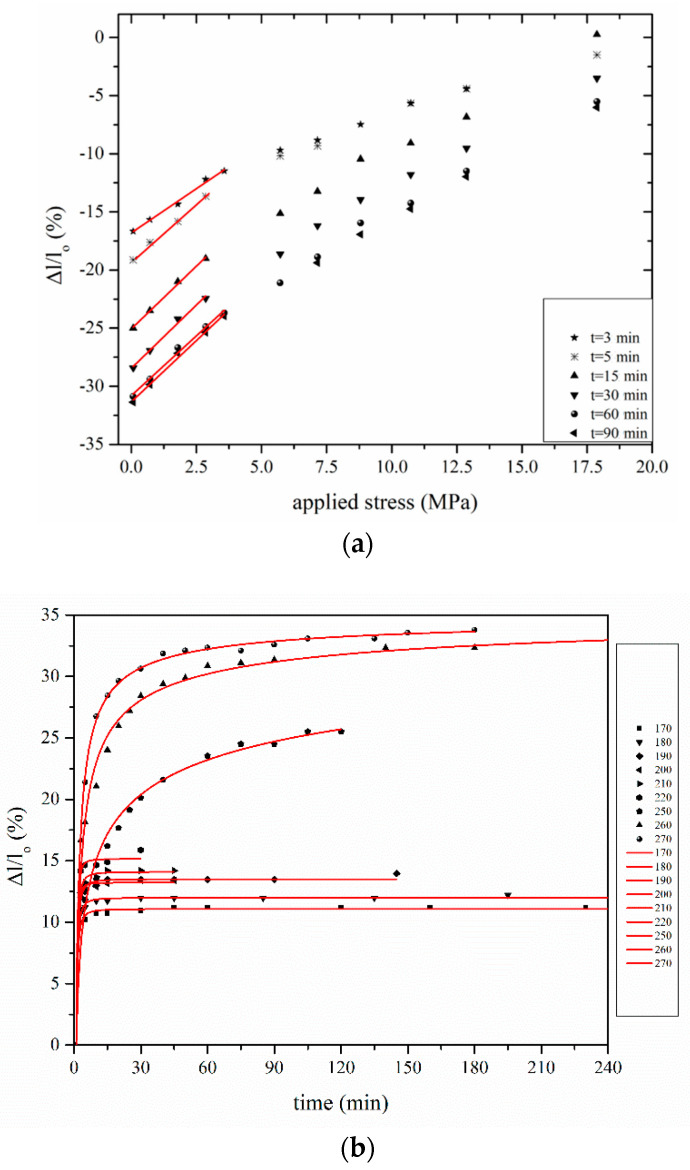
(**a**) Isochronous change of length during isothermal stabilization. (**b**) Entropic shrinkage versus treatment time for YTD PAN fiber at different temperature.

The non-linear thermoplastic behavior of PAN during stabilization can be clearly seen by plotting isothermal and isochronous change of length versus the applied stress (Figure 4a). However, the lower part of the curves (i.e., for applied stress up to 5 MPa) shows a linear dependence between shrinkage and tension, indicating that PAN can be considered as a linear viscoelastic material at this region [44]. The intercept of the straight line represents the maximum value of shrinkage achieved under “free shrinkage” conditions (i.e., with no applied stress, *σ* = 0) for that temperature–time combination [1]. This value is identical to entropic shrinkage (ES), which is connected to the fibers irreversible entropic recovery (since the macromolecules of PAN revert from a highly oriented structure to the random coil configuration) [31,45]. An increasing trend of ES versus temperature was proven, reaching a plateau value by observing the evolution of stabilization versus time.

In Figure 4b, ES-time curves are presented. Fitting of ES-treatment time points was performed using non-linear regression, with the best approximation achieved when a two-parameter power equation was used:

(1) $${\left(\frac{\Delta l}{l_{o}}\right)}_{ES}{=ES}_{\max}\cdot {(1-t}^{-b}{),\;t<t}_{onset}$$

**Figure 5:**
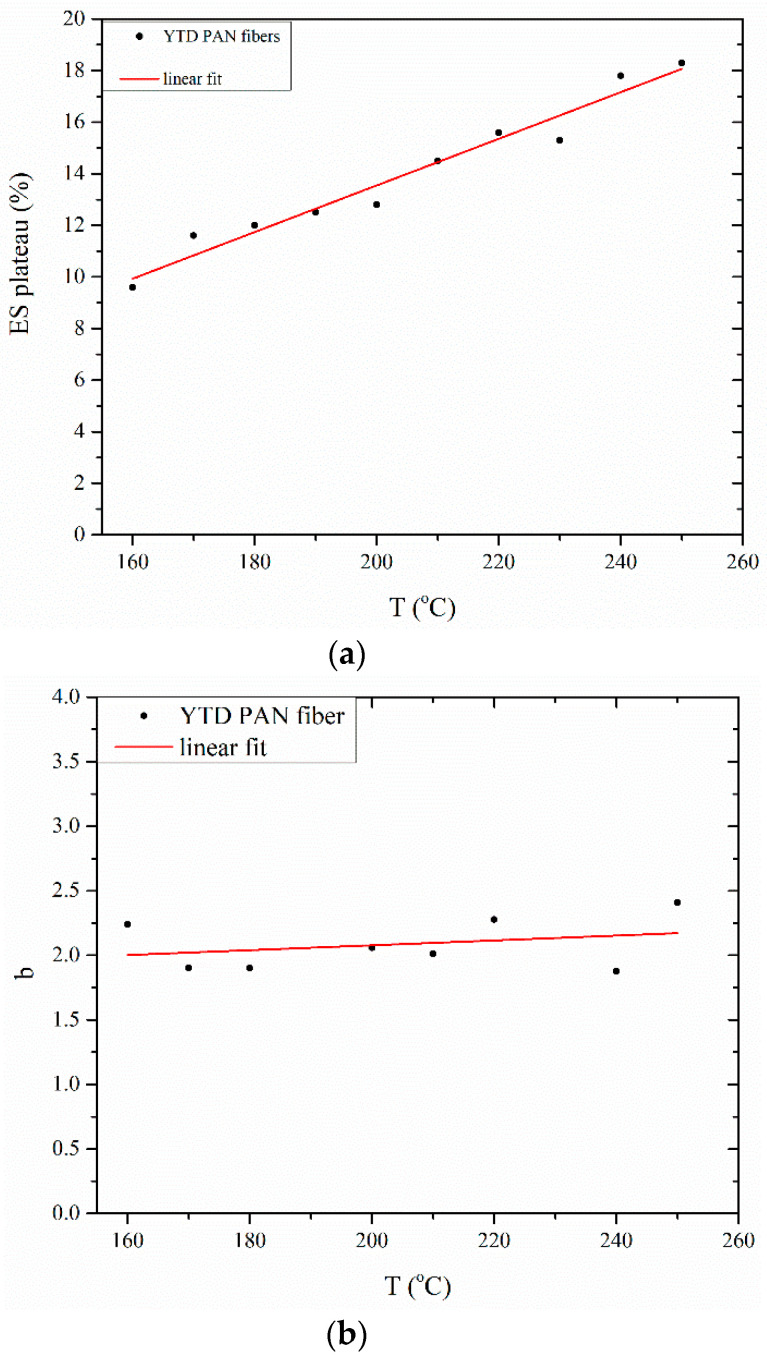
(**a**) Entropic shrinkage (*ES*) plateau parameter and (**b**) exponent b of Equation (1) in relation with stabilization temperature.

In Equation (1) *ES*_max_ corresponds to the maximum-value of *ES* as already mentioned, while b value is used to describe the time-dependence of *ES*. Figure 5 shows that the maximum *ES* value generally increases with temperature. This is attributed to increased chain mobility as a result of stabilization reactions propagation, which reduce nitrile polar interactions, and intermolecular cohesion forces. PAN polymer contains segments that are characterized by their crystallinity, namely a paracrystalline and an amorphous phase [23,46]. The ordered phase content was minimized at 150 °C (under free shrinkage conditions) [24], while intermolecular interactions are higher in the amorphous than the ordered state [46]. Thus, an increase in the amorphous phase fraction seems to obstruct *ES* at 150 °C. Even more, the evolution of *ES* during stabilization may be influenced by the different shrinkage rates between the amorphous and crystalline regions of PAN [1,10]. In the present study, shrinkage was varied between 10 and 20% with increase in stabilization temperature. In Figure 5, the values of parameter b are approximately equal to 2.09 in case of YTD PAN fibers.

### 3.2. Deconvolution of Creep Effect

**Figure 6:**
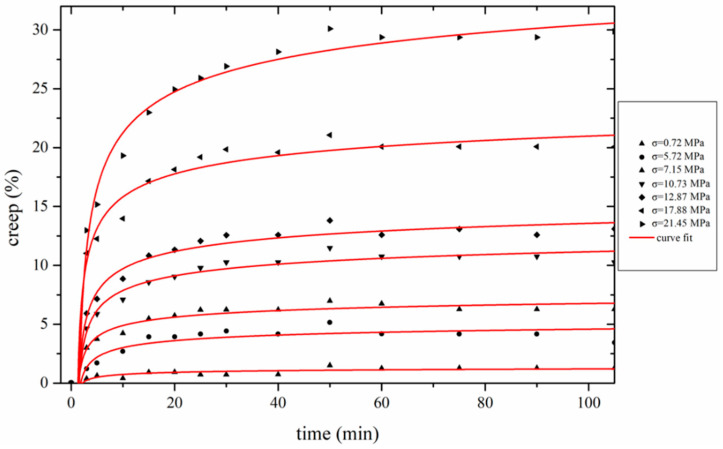
Creep curves for isothermal stabilization of YTD PAN fibers at various applied stress.

The thermomechanical evolution of the polymeric PAN fibers can be described using fundamental models. Burgers model does effectively describe influence of the elastic, the viscoelastic, and the viscoplastic contribution and is used to describe the creep behavior. The elastic terms correspond to recoverable creep and plastic to permanent plastic deformation [47]. In the case of thermal oxidative treatment of PAN fibers, due to the lack of length recovery, it was assumed that permanent change in length occurred, hence, the creep can be described as a viscoplastic flow. Following the determination of *ES*, the creep was calculated by removing the *ES* contribution to length change (Figure 6). It can be observed that PAN fibers do not behave as a linear viscoelastic polymer, when high stress is applied during stabilization. Even though there are several possible approaches that could be used for investigating this non-linear creep behavior in accordance to Eyring approach, the proposed model is shown in Equation (2) [22,48]:

(2) $${\left(\frac{\Delta l}{l_{o}}\right)}_{creep}=A\cdot (c_{T}-a_{T}\cdot T)\cdot (a_{m}\cdot \sigma -c_{m})\cdot t\cdot \exp\left(-\frac{E_{a}}{RT}\right)\cdot \exp\left(-\frac{\delta \cdot \sigma }{2k_{B}T}\right)\;$$

where, 

*A*: pre-exponential factor (sec^−1^·K^−1^)

*T*: treatment temperature (K)

*t*: duration of the treatment (sec)

*E_a_*: activation energy of creep (kJ∙mol^−1^)

*a_T_*: slope related to temperature

*c_T_*: constant related to temperature

*R*: gas constant (8.314 J·K^−1^∙mol^−1^)

*σ*: stress applied on the fibers (MPa)

*δ*: activation volume (Å^3^)

*k_B_*: Boltzmann constant (1.381 × 10^−23^ J·K^−1^)

*a_m_*: slope related to applied stress

*c_m_*: constant related to applied stress

This expression is adapted from a similar equation that was firstly proposed for modeling the shrinkage of PAN fibers during their thermomechanical treatment and it has been derived from the Eyring model that describes the flow of solids as an activation energy-driven process [22,48].

The required stress for preventing fiber shrinkage can be obtained using Equation (2), considering steady state of creep during stabilization, and for adjusting the applied tension in order to ensure that physical damage of orientation and crystallinity is prevented [24,49].

Equation (2) contains several structural parameters that should be calculated from the experimental results before its implementation; their calculation will be presented below.

#### 3.2.1. Determination of Creep Activation Volume *δ*

**Figure 7:**
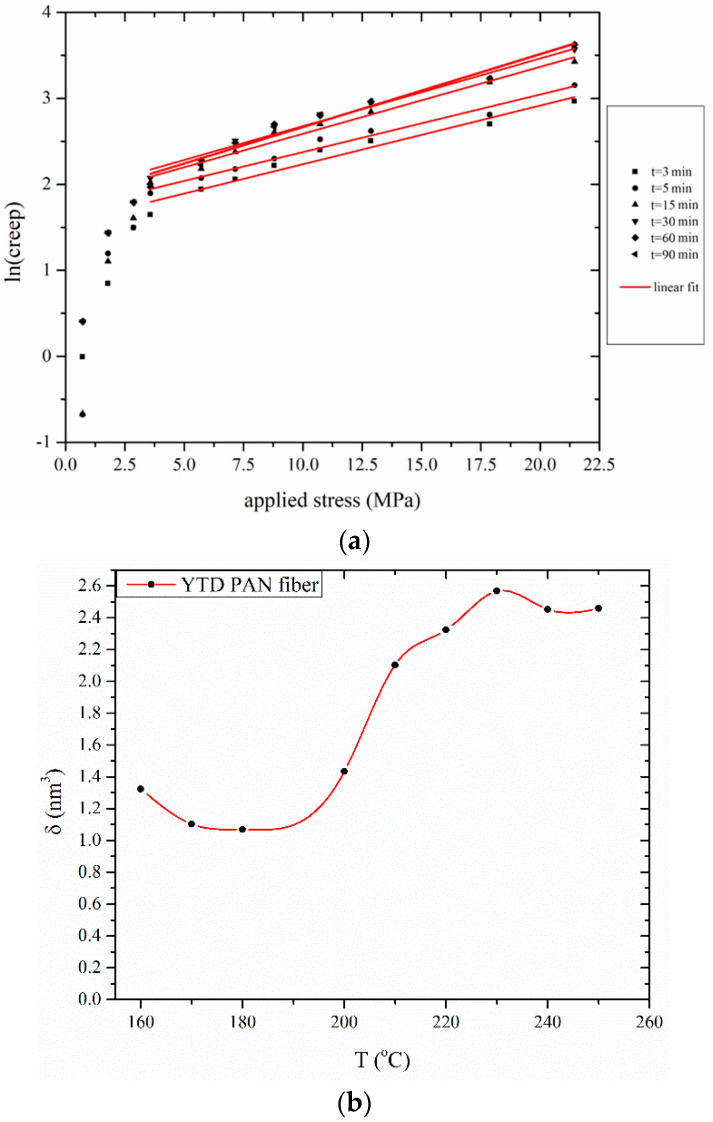
(**a**) Isochronous plot of creep logarithm vs stress during stabilization of YTD PAN fibers and (**b**) dependence of creep activation volume (*δ*) on stabilization temperature for Fibers A and B.

Initially, the activation volume *δ* can be estimated using isochronous plots of creep logarithm versus applied stress (*σ*) for a given temperature (Figure 7). In this occasion Equation (3) is transformed as:

(3) $$\ln{\left(\frac{\Delta l}{l_{o}}\right)}_{creep}=\ln(A\cdot t)+\ln(c_{T}-a_{T}\cdot T)+\ln(a_{m}\cdot \sigma -c_{m})-\frac{E_{a}}{RT}-\frac{\delta \cdot \sigma }{2kT}$$

In Equation (3), the parameter: [$\ln(c_{T}-a_{T}\cdot T)+\ln(a_{m}\cdot \sigma -c_{m})$] can be considered as constant. It should be noted that creep below 5 MPa (critical value), was excluded, as the behavior changes to linear viscoelastic.

*δ* was determined for each parameter of time and temperature of stabilization parameterization; for a fixed temperature *δ* is practically independent of treatment time. The aforementioned trend was anticipated considering the creep elongation mechanism, derived by displacement and increase in distance between basic molecular units in PAN macromolecule. The dependence of *δ* on temperature is shown in Figure 7b. More specifically, at low temperature the onset time for dehydrogenation and/or oxidation is several hours long, which is in accordance to visual observations of fiber color change [22]. Should dehydrogenation be the case, it could have been surmised that the importance of the reactions would had increased by increasing temperature (i.e., leading to even higher *δ* values). Oppositely, chemically-induced changes are minimal in this temperature range. This fact indicates that any change should be attributed to the ordered regions, since it has been demonstrated that low-temperature stabilization under tension increases orientation and the extend of the ordered phase (L_c_) [23]. Between 150 and 170 °C L_c_ is increased from 90.3 to 111.3 nm. This increase is originated from structural changes induced by the increase of the inter-chain spacing of the ordered regions from about 6.14 to about 6.17 nm [50]. The enlargement of the ordered phase resembles the “stress-induced crystallization”. It is supposed that this increase in L_c_ may reduce segmental mobility, which as an effect decreases *δ* value.

It is worth pointing out on the aforementioned (and similar) decrease in *ES* plateau values (Figure 5), however, in the latter case the decrease is demonstrated at different temperature (150 °C, compared to 160 °C for *δ*). Bashir [46] attributed this to the glass–rubber transition of the amorphous phase of PAN. Also, one can correlate this deviation to the “stress-free” *ES* (“free shrinkage” conditions), and the dependence on the composition in amorphous phases. On the other hand, the values of *δ* include the stress effect, and thus the observed transition may be influenced by the ordered phases. It is also worth noticing in another work, that thermomechanical behavior of PAN fibers lead to a similar conclusion [42]. However, the most possible scenario is that this temperature discrepancy of the two parameters are dependent on copolymer composition [49,51]. The decrease of *δ* above 220 °C could possibly be attributed to the effect of chemical reactions on fiber structure. Dehydrogenation reactions create conjugated structures on the polymer backbone, leading to increased rigidity and reducing the segmental mobility [31].

#### 3.2.2. Evaluation of Creep Activation Energy

For the calculation of activation energy, the treatment conditions should be defined, where the fibers have the same degree of creep when subjected to the same applied load. These points can be detected in the isostress plot of the creep vs. time; afterwards, they are plotted in the form of [ln(t*T) vs. (1/*T*)]. Then, the activation energy can be calculated from Equation (4) (C representing a constant):

(4) $$\ln\left(t\cdot (c_{T}-a_{T}\cdot T)\right)=\frac{E_{a}}{R}\cdot \frac{1}{T}+C$$

An average value of *E_a_* is calculated as 335 kJ/mol. This value is rather high and means that the effect of the term: [exp(−*E_a_*/*RT*)] on the creep is not as important as the other parameters in Equation (2). This possibly also explains the already mentioned insensitivity of the intercept in the isochronous [ln(*creep*)−*σ*] plots (Figure 7a) to the temperature.

**Figure 8:**
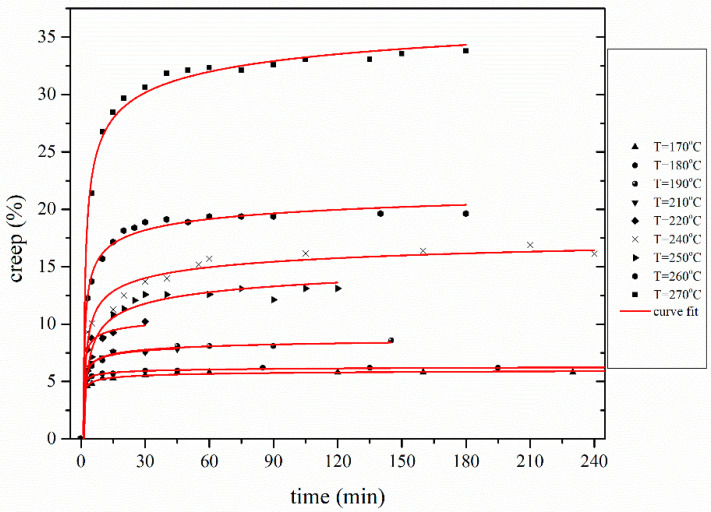
Creep elongation behavior during stabilization at isostress conditions at 12.87 MPa for YTD fiber.

Figure 8 shows the fitting of the proposed model with the corresponding experimental creep–time data sets—a good approximation is achieved.

### 3.3. Modeling of Chemical Shrinkage (CS)

The chemical shrinkage (*CS*) is induced as a result of cyclization propagation the thermal oxidative treatment. Specifically, intra-molecular cyclization causes considerable decrease of the length of the PAN macromolecules. The initiation of *CS* requires an incubation time. It has been shown that dehydrogenation and oxidation reactions take place before the onset of cyclization, and do not affect the thermomechanical properties of PAN fibers [31]. Since apart from cyclization no other reaction induces chemical shrinkage, the chemical properties of PAN fibers during stabilization are described by modeling chemical shrinkage and cyclization, that induce stereochemical rearrangement. There are numerous references in literature that evaluate stabilization progress by the degree of cyclization in order to optimize the mechanical properties after carbonization [52].

The required duration for the onset of *CS* is termed as cyclization onset time (*t_onset_*). In the treatment region below the onset of *CS*, oxidation and dehydrogenation affect only marginally the length of PAN fibers (even though they do affect the structure of the PAN fibers) [53,54]. It has been found that the *CS*–time curves can be described by an exponential relation [54,55]:

(5) $${\left(\frac{\Delta l}{l_{o}}\right)}_{CS}={\left[CS\right]}_{\max}\left(1-e^{-k\cdot t_{R}}\right)\;t\;>\;t_{onset}$$

**Figure 9:**
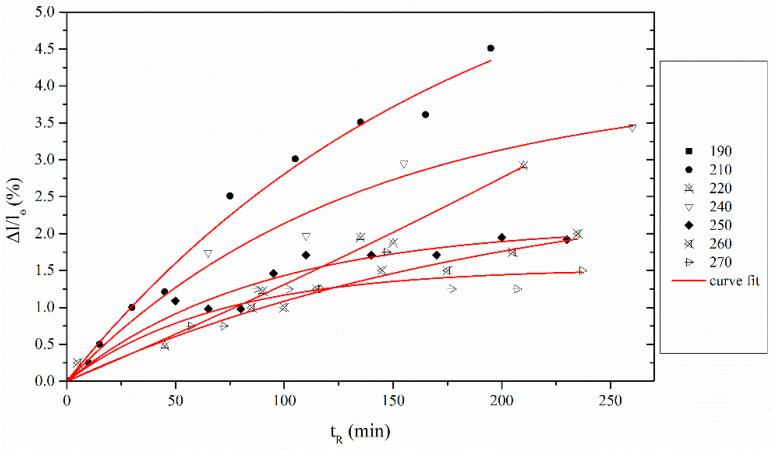
Chemical Shrinkage fitting using stabilization model versus reaction time t_R_.

This expression can be directly used to examine whether nitrile cyclization is described by first order kinetics [31], which was also confirmed for the PAN fibers investigated (Figure 9). Parameter [*CS*]_max_ in Equation (5) is temperature-specific and symbolizes the maximum extent of *CS*, and it is possible to be used in the estimation of the intra- and inter-molecular nitrile cyclization ratio.

The parameter k is corresponding to nitrile cyclization rate and can be used for determining the kinetics of the cyclization reaction. For YTD PAN fibers the cyclization activation energy was calculated at 131.6 kJ/mol. As reported in the literature, cyclization *E_a_* is in the region of 100–150 kJ/mol, while any deviation could be attributed to the different comonomers used in PAN various copolymers [31,56]. The *CS* plateau increased gradually from 8% up to 14%, indicating that cyclization proceeds mainly through inter-molecular reactions [30,31].

(6) $$\ln(t_{onset})=\frac{E_{a}}{R}\times \frac{1}{T}-\ln A_{o}$$

The values of *E_a_* and *k_o_* were measured for YTD PAN fibers at 140.3 kJ/mol and 0.476 s^−1^.

**Figure 10:**
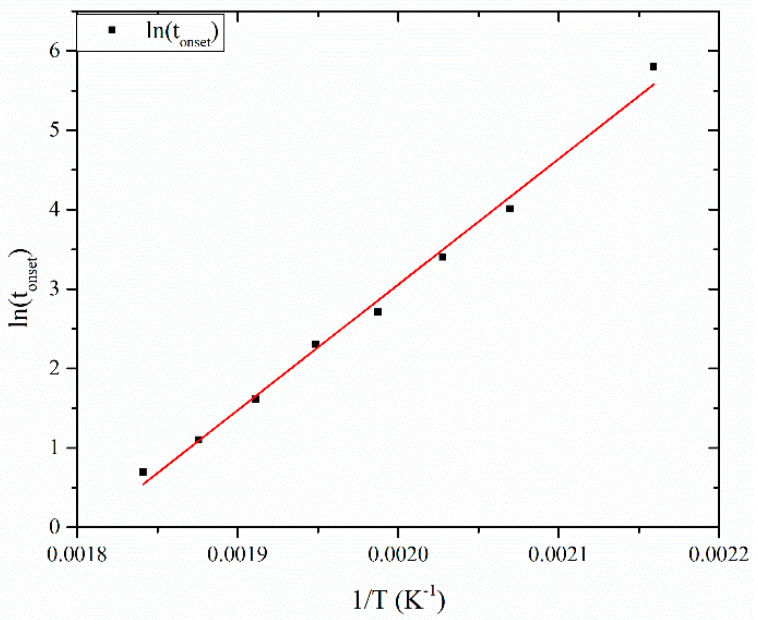
Arrhenius plot of *t_onset_* versus stabilization temperature.

The final aspect of the model is *t_onset_*. Figure 10 shows that *t_onset_* has an Arrhenius-type dependence on temperature. This parameter actually represents the treatment conditions, where cyclization reactions are initiated and the time when the conversion *α* is zero and d*α*/dt is positive.

### 3.4. Fourier Transform Infrared Spectroscopy

**Figure 11:**
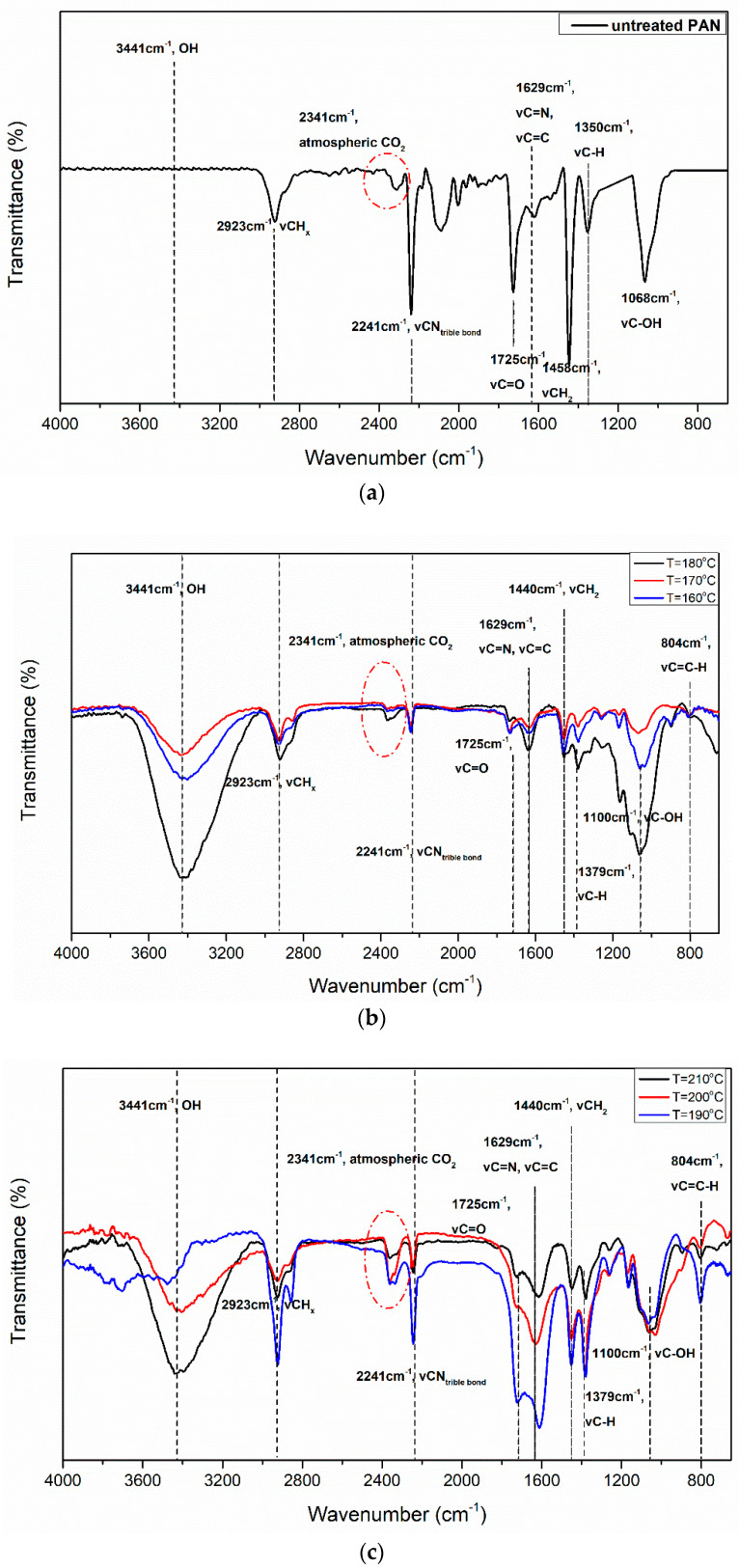

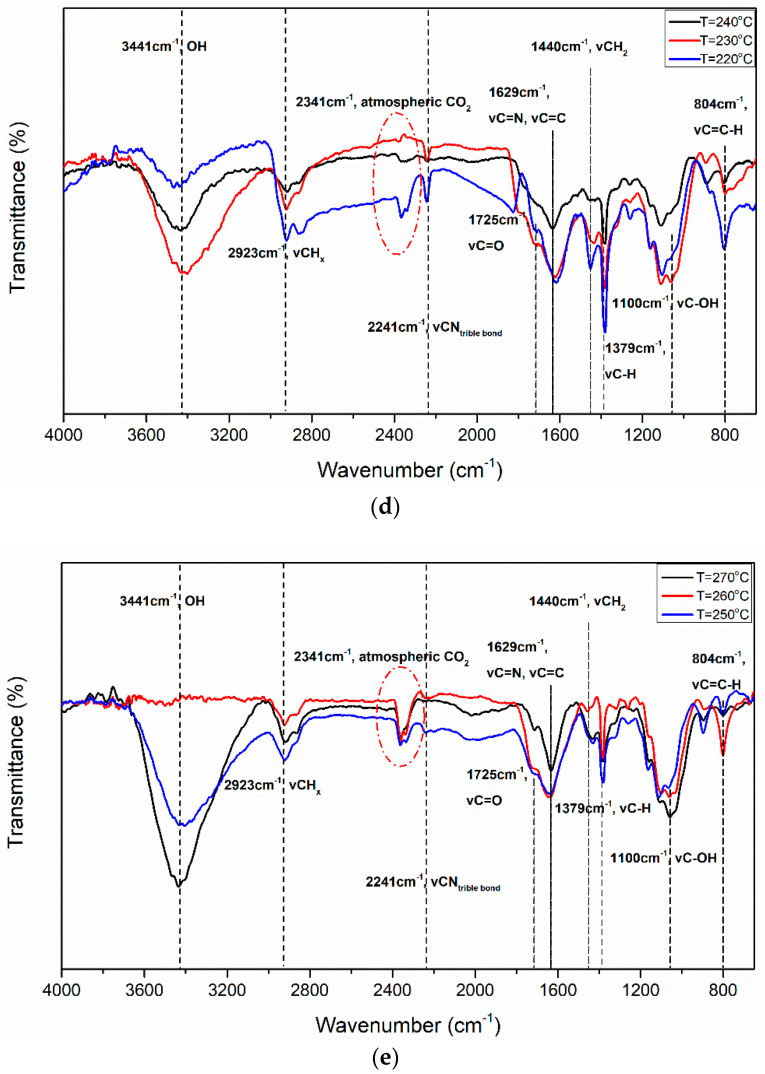
Fourier Transform Infrared Spectroscopy (FTIR) spectra of virgin and stabilized fibers in range of (**a**) untreated, (**b**) 160–180 °C, (**c**) 190–210 °C, (**d**) 220–240 °C, and (**e**) 250–270 °C. In each case, treatment duration was 4 h.

Figure 11 shows the FTIR spectra of several oxidatively treated PAN fibers. According to literature, applied load does not affect chemical structure of treated fibers [22,31], and thus stabilized fibers were characterized independently of the applied load.

It can be seen that, by increasing intensity of treatment (either the temperature or the duration), the peaks at 2940 and 2240 cm^−1^ are decreased (attributed to methylene and nitrile groups, respectively), due to dehydrogenation and cyclization reactions, respectively. Peaks approximately at 1740 and 1600 cm^−1^ are attributed to the carbonyl and the conjugated double bonds are progressively formed as expected, with simultaneous decrease of the methylene groups (which can be better observed from the peak at 1446 cm^−1^ rather than 2924 cm^−1^) [7,8,11,13,16,22,24,29,57,58]. By comparing the spectra at 150 °C and 200 °C, a higher reduction of methylene groups than of the -C≡N groups is observed, indicating that in the latter case dehydrogenation progressed to higher degree. The peaks at 1382 and 1219 cm^−1^ are mainly associated to C−H, which should not be detected in thermally stabilized PAN fibers due to dehydrogenation.

The peaks in the FTIR spectra are summarized in Table 1. Due to the use to KBr pellet technique, several peaks can be used for quantitative analysis, in order to monitor the reaction progress. Cyclization index (*CI*) and nitrile to methylene ratio (*r_nm_*) are used:

(7) $$CI=\frac{I_{c}}{I_{C}+I_{n}}$$

(8) $$r_{nm}=\frac{{\left(\frac{I_{n}}{I_{m}}\right)}_{treated}}{{\left(\frac{I_{n}}{I_{m}}\right)}_{pristine}}$$

where *I_c_*, *I_n_*, and *I_m_* are the intensities of peaks at 1600 (conjugated double bonds), 2240 cm^−1^ (nitrile groups) and 2940 cm^−1^ (methylene groups), respectively.

Using Table 1 to investigate the chemical transformations during the treatment of the fibers at different temperatures, the FTIR spectra in Figure 11 showed a progressive reduction of nitrile bonds, along with a reduction of the methylene groups of the macromolecular backbone. Simultaneously, as the temperature is elevated, the formation of C=N and C=C conjugated double bonds progresses, as observed by the growth of the peak in the region of 1600 cm^−1^, along with the carbonyl groups (C=O) at 1730 cm^−1^, and of the C-OH units at 1100 cm^−1^, which were formed due to oxidation reactions during stabilization; moreover, the appearance of a peak at approximately 800 cm^−1^ can be ascribed to the C=C-H footprint of stabilization process at 806 cm^−1^. In order to quantifiably evaluate the stabilization, Equations (6) and (7) are used, and the results are summarized in Table 2.

Oxidative treatment at low temperatures (i.e., 160 and 170 °C) showed low cyclization yield. However, the appearance of a peak at 1640 cm^−1^ is connected to conjugated double bond structures formation. The values of *r_nm_* indicate that these structures are the result of the dehydrogenation reactions. By increasing the treatment temperature above 190 °C, the value of *CI* increased due to extensive cyclization. Generally, when the treatment is in the region 200–210 °C the values of *r_nm_* exceed 1, indicating that both dehydrogenation and cyclization reactions contribute to ladder polymer formation. The gradual decrease of the *r_nm_* values in the temperature region 200–270 °C indicates that increasing contribution of the cyclization reactions to the formation of conjugated double bond structures as the treatment temperature increases. Above 240 °C, CI reaches high values indicating that cyclization reactions are almost complete. During high-temperature stabilization (i.e., between 250 and 270 °C) a large methylene peak, a weak peak of carboxyl groups at 1730 cm^−1^, and a peak for the conjugated double bonds at 1630 cm^−1^ were detected. As it is implied, in this temperature region oxidation occurs in a low extend, probably due to the slow diffusion of oxygen in the fiber. Moreover, oxygen may initiate an alternative reaction pathway towards the formation of active centers for cyclization, thus the preferential initiation of the cyclization reaction acts competitively to the oxidation reactions [13,23,24,37].

### 3.5. Raman Spectroscopy

In order to investigate the evolution of the microstructure and the formation of ordered graphitic planes, Raman spectroscopy was performed on Fiber B specimens. These fibers were treated isothermally at 160–270 °C for 4 h duration under 300 g of applied force. The samples were fixed on a microscope glass slide in order to have the best focus on the analysis point. It was possible to detect the Raman signal only in samples treated at temperatures above 200 °C. In these samples, an extremely low intensity of signal in the 2D region was observed. In addition, the shape of the signal did not show a peculiar behavior and the introduced measure uncertainty by the baseline correction procedure was high. For these reasons the measurement was performed in static scan mode with center at 1550 cm^−1^. Laser power started from 0.06 mW, and reached 3 mW or 50% of the maximum available power of 6 mW.

**Figure 12:**
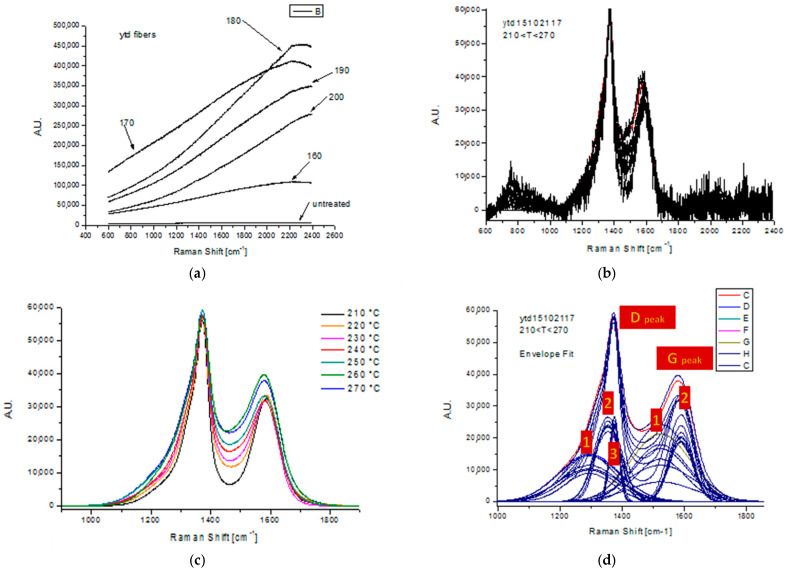
Raman spectra of thermally treated fibers: (**a**) isothermally treated at temperature between 160–200 °C. Isothermally treated at temperature between 210–270 °C, (**b**) after Baseline correction and subtraction, and (**c**) after noise reduction and D-peak normalization. In (**d**) the fitting process in the entire spectrum is demonstrated to measure the D-and G-peak area as presented in Table A1. Raman Data Tables.

As can be observed in Figure 12a, untreated and thermally treated samples in the range 160–200 °C presented a similar spectroscopic behavior regarding the lack of peak detection. To avoid charge-coupled device (CCD) sensor saturation, all the samples were measured at low power of the laser source, from 0.006 mW to 0.06 mW. All the samples showed an intense fluorescent signal without any trace of D and G peak Raman activity. The lack of any Raman activity indicates the absence of cyclized structures, but the strong fluorescent signal can be accredited to the presence of non-cyclized organic compounds. Any comparison can be related to carbon fibers, where Raman characterization is essential to characterize the carbonization and graphitization progress, where the turbostatic graphitic structure formation is completed crystalline order [59,60].

The Raman spectra of thermal treated samples in the region of 210–270 °C are presented in Figure 12b. Fibers treated at temperatures above 200 °C exhibited Raman activity similar to the stabilized PAN fibers, an indirect indication of oxidation progress [11,24,34]. In this case, the presence of Raman activity in both the D and G regions can be seen (along with a strong fluorescent signal). These fibers contain ladder polymer structures and have shiny black color, thus, a greater laser power could be applied to analyze the samples (in order to avoid the saturation of CCD sensors). The laser source power ranged from 10 to 50% of maximum potential. The Raman spectra of Figure 12b were normalized on the D peak at 1372 cm^−1^ and cleared by noise reduction; the final results are presented in Figure 12c. For further quantitative analysis of structural transformations during the treatment, the curves of the Raman spectra stabilized fibers were fitted using a number of peaks that describe the entire signal, as seen in Figure 12d.

The initial PAN fibers and those treated below 200 °C are Raman inactive. A different trend is presented at higher temperatures, where cyclization seems to have altered the structure of the fibers [61]. Looking the peak fitting in Figure 12d, it can be clearly seen that the D and G regions are fitted with five different Gaussian components, three for the D peak and two for the G peak. The D peak is described by two components centered at 1354 and 1375 cm^−1^, respectively. This exhibits a small intensity variation with the treatment changes of the treatment temperature and the third component (around 1290 cm^−1^) shows higher variation. The G peak has a component around 1590 cm^−1^ not significantly affected by treatment temperature and a second component at 1520 cm^−1^ that increases as the treatment temperature increase [51,62,63]. In general, as temperature is increased the ratio I_D_/I_G_ tends to decrease, due to the enrichment of the fibers with turbostatic graphite structures (i.e., the G peak), due to the effect of cyclization and aromatization reactions, and thus stabilization treatment at elevated temperature is proved to be more effective [64,65,66]. Amplitude, center, and width for each component are reported Appendix A (in Table A1. Raman Data Tables).

### 3.6. Thermal Analysis

**Figure A1:**
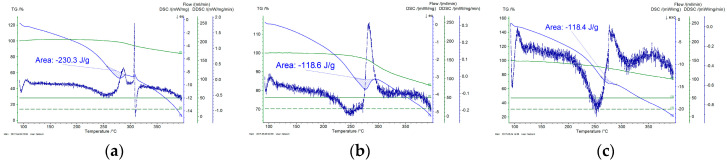

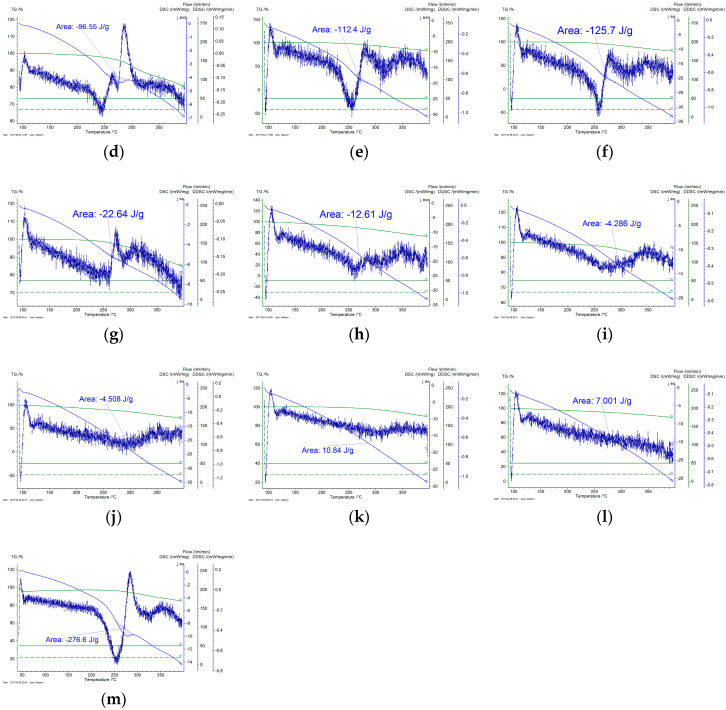
DSC-TGA-DTG analysis of PAN fibers stabilized (**a**) 160 °C, (**b**) 170 °C, (**c**) 180 °C, (**d**) 190 °C, (**e**) 200 °C, (**f**) 210 °C, (**g**) 220 °C, (**h**) 230 °C, (**i**) 240 °C, (**j**) 250 °C, (**k**) 260 °C, (**l**) 270 °C, and (**m**) untreated in air atmosphere.

The results of the thermal analysis in air atmosphere are presented in Appendix A (Figure A1) and are summarized in Table 3.

**Figure A2:**
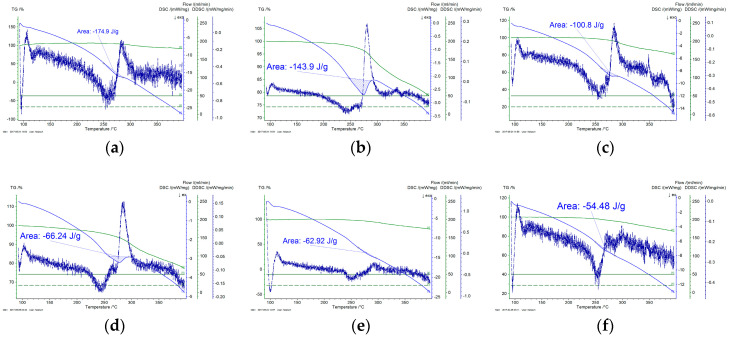

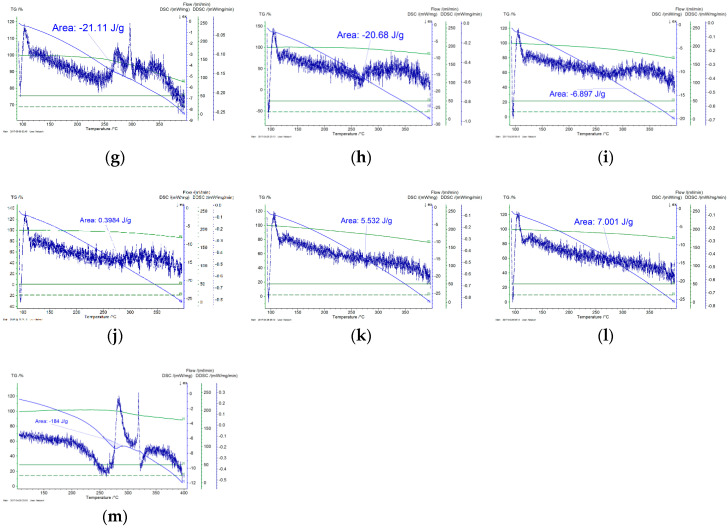
DSC-TGA-DTG thermal analysis of stabilized PAN fibers at (**a**) 160 °C, (**b**) 170 °C, (**c**) 180 °C, (**d**) 190 °C, (**e**) 200 °C, (**f**) 210 °C, (**g**) 220 °C, (**h**) 230 °C, (**i**) 240 °C, (**j**) 250 °C, (**k**) 260 °C, (**l**) 270 °C, and (**m**) untreated, tested in inert N_2_ atmosphere.

The results of the thermal analysis in nitrogen atmosphere are presented in Appendix A (Figure A2). The measured heat released is due to the exothermic, intra- and inter-molecular cyclization, the only occurring action under such conditions (since dehydration dehydrogenation and oxidation reactions are triggered by the presence of O_2_) [1,6,11,13,34,49,67]. Confirmation that only a single event occurs is obtained by TGA (where a bending point is observed in DTG), while the DSC exotherm is accompanied by minor weight loss, a behavior characteristic of cyclization reactions [29]. The results of this analysis are presented in Table 4.

### 3.7. Comparison of the Cyclization Yield Derived from DSC, FTIR, and Chemical Shrinkage Model

**Figure 13:**
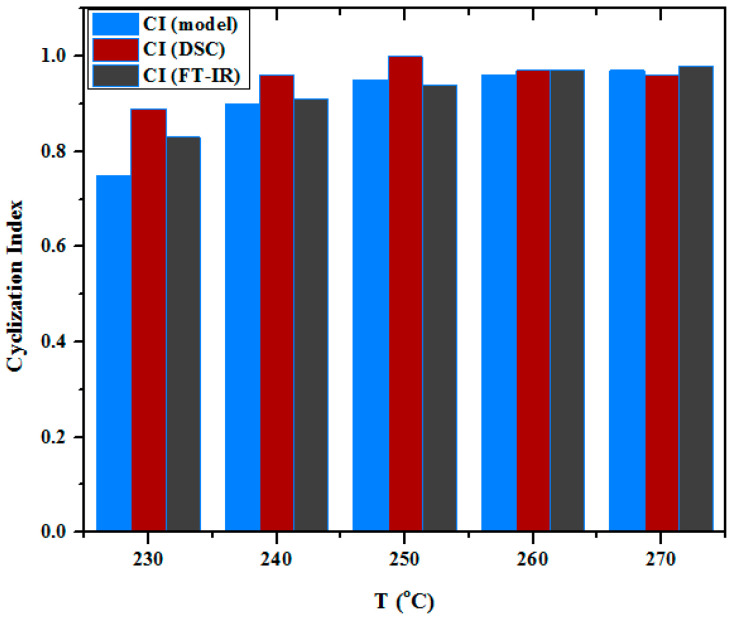
Comparison of estimations for the cyclization progress derived from three different methods: Chemical shrinkage model, DSC, and FTIR.

The enthalpy measured via DSC can be used for determining the progress of the cyclization through the Cyclization Index (CI) [41]. The results obtained by DSC can be compared with those acquired from the FTIR measurements and with those derived from the model of chemical shrinkage [1,8,29,34,51]. The results of the different approximations are summarized in Figure 13. It is clear that the three methods yield similar results for the progress of the cyclization. As a general trend, DSC- derived CI tend to have somewhat higher values. This is probably attributable to the limitations of DSC as a measurement method, namely, due to the definite heating rate, part of the heat evolved at high temperatures diffuses and is not accounted for, i.e., the residual exotherm is underestimated. The differences between the CI values estimated from the chemical shrinkage model and from the FTIR decrease as the treatment temperature increases. This effect could be attributed to the effect of dehydrogenation. Namely, the CI derived from chemical shrinkage takes into account only the effect of the nitrile cyclization reactions, whereas the CI from FTIR accounts for all conjugated structures (independent of their origin). It seems that the fibers treated at 230 °C contain a considerable amount of linear conjugated structures in their structure, which is the end product of dehydrogenation reactions. Gradually, as the treatment temperature increases, the CI values increase and their differences diminish. The latter phenomenon is a clear indication for the domination of the nitrile cyclization reactions and the concurrent marginalization of the backbone dehydrogenation reactions.

### 3.8. Investigation of Cross-Section Circularity after Thermal Treatment

**Figure 14:**
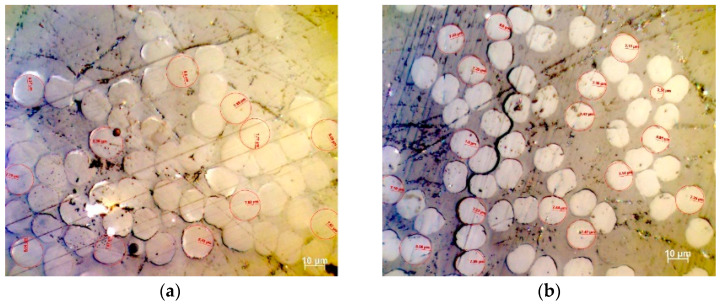

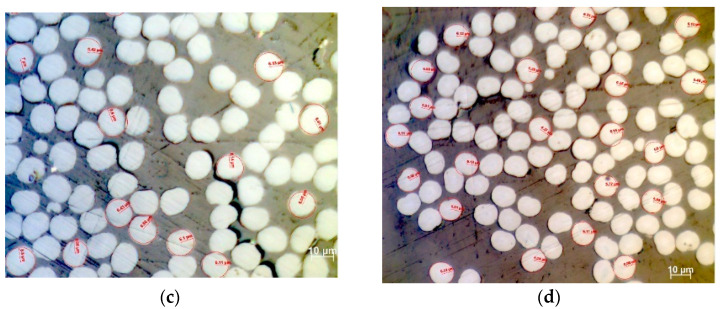
Optical Microscopy of stabilized PAN fibers at temperatures of (**a**) 160 °C, (**b**) 210 °C, (**c**) 230 °C, and (**d**) 260 °C.

The cross-section area of treated PAN fibers at 160, 210, 230, and 260 °C (that were observed by optical microscopy) was pointed out that the chance of cross-sectional shape alternation was almost 1:1, so that when a monofilament was observed, the possibility to be of circular area is almost the same as to obtain a different shape (Figure 14). In order to prove the effect of cross-sectional shape alternation in the estimation of the total fiber cross-sectional area, a thorough study of the stabilized fibers surface was conducted. In each case, three characteristic areas were measured, while at least 40 monofilaments were measured for initial and treated fibers, considering the features of radius and area in order to obtain statistically correct results. A mean value was estimated along with the standard deviation, while the measurements in case of deformed fibers were obtained using ImageJ. An equal cyclic radius (ECR) was estimated- (or equivalently the diameter, ECD) [1], along with a mean value, and the standard deviation were determined. The results are summarized in Table 5 below.

As presented in Table 5, the cross-section of deformed fibers exhibits a decreasing trend with temperature. However, estimation of mean area permitted the estimation of the importance of cross-section variation. For all cases, the variation in PAN monofilament radius and the derived total area was in the limits of standard deviation, and more specifically approaching the lower limit.

## 4. Conclusions

Following extensive characterization and mapping of the stabilization process, a model was introduced to control the process. This model may be applied to other polymeric fibers that are thermally processed via stabilization, such as polyaramide, lignin, and Rayon fibers. A common feature of these fibers with PAN is the thermoset structure obtained after stabilization, which is introduced by the cyclization reactions. Exploitation of the model should provide a shortcut for process optimization regarding the time-and energy-demand, without getting into the complex chemistry of stabilization. The following remarks define the new insights gained in this study:

1. Cyclization yield can be accurately calculated at high temperature by the model implementation, wherein macroscopic change in length was involved. The results were further confirmed by FTIR and DSC-derived reaction yields.
2. Control of cyclization yield via adjustment of stabilization variables could optimize mechanical properties after carbonization via the optimization of ladder polymer structure.
3. A further gain is the applicability of this model for oxidative stabilization at both batch and continuous processing plants, as the employed values of temperature, tension, and residence time are taken into consideration to measure cyclization index.
4. Using FTIR and Raman spectroscopy, it is feasible to observe the evolution of chemical structure and ladder polymer formation, using aromatic index, and G-peak activity.
5. Thermal analysis provided additional insight regarding the progress of cyclization and the overall progress of stabilization reaction. It was also confirmed by FTIR analysis that dehydrogenation is the basic reaction that occurs below 200 °C. Rate of both cyclization and oxidation is increased at higher temperatures.
6. Raman confirmed the structural transformation of linear PAN molecule to ladder polymer, where the formation of graphite-like sp^2^ planes is evidenced as a result of stabilization at temperatures above 200 °C.
7. Optical Microscopy demonstrated that thermal treatment had minor effect in fibers microstructure, as proved by the observation of the area and the circularity of thermal treated PAN fibers.

Consequently, in order to obtain a sufficiently stabilized PAN fiber, the fiber should be treated at temperature higher than 200 °C. It is expected that by involving a multistage process, the yield of stabilization process could be increased. 

## Figures and Tables

**Table 1:** Major peaks to be characterized in FTIR spectra.

Wavenumber (cm^−1^)	2924	2239	2107	1725	1658	1583	1446	1357 & 1262	1163	1097 & 1064	804
Bonds	CH_x_	C≡N	C=NH	C=O	C=C asymmetral vibration	C=C,C=N	Tensile vibration CH_2_	C-H vibration	C-O	C-OH	=C-H
Major FTIR peaks before stabilization	√	√	√	√	×	×	√	×	√	×	×
Major FTIR peaks after stabilization	√	↓	√	↑	√	√	↓	√	√	√	√

**Table 2:** Values of treatment r_nm_ and cyclization index (CI) indexes measured from FTIR spectra via Lambert–Beer Law.

Stabilization Temperature (°C)	*CI*	*r_nm_*	Cyclization	Dehydrogenation
160	0.43	2.50	low	High
170	0.44	1.91	low	Medium
190	0.65	2.24	mid	High
200	0.64	2.97	mid	High
210	0.64	1.84	mid	High
230	0.83	0.99	high	High
240	0.91	0.74	high	High
250	0.94	0.44	high	High
260	0.97	0.41	high	High
270	0.98	0.11	high	High

**Table 3:** Results of thermal analysis of oxidatively stabilized PAN fibers.

Stabilization Temperature (°C)	ΔH_total_ (J/g)	Stabilization Index (SI)
---	276.6	0.0%
160	230.3	16.7%
170	118.6	57.1%
180	118.4	57.2%
190	96.6	65.1%
200	112.4	59.4%
210	125.7	54.6%
220	22.6	91.8%
230	12.6	95.4%
240	4.3	98.4%
250	4.5	98.4%
260	10.8	96.1%
270	19.1	93.1%

**Table 4:** Results of thermal analysis of stabilized ΡAΝ fibers in inert atmosphere.

Stabilization Temperature (°C).	ΔH_cyclization_ (J/g)	Cyclization Index (CI)
---	184	---
160	174.9	4.9%
170	143.9	21.8%
180	100.8	45.2%
190	66.2	64.0%
200	62.9	65.8%
210	54.5	70.4%
220	21.1	88.5%
230	20.7	88.8%
240	6.9	96.3%
250	0.4	99.8%
260	5.5	97.0%
270	7	96.2%

**Table 5:** Synopsis of alternation of PAN cross-sectional area investigation results through optical microscopy (OM).

Stabilization Temperature (°C)	160		210		230		260	
**Software**	**OM**	**ImageJ**	**OM**	**ImageJ**	**OM**	**ImageJ**	**OM**	**ImageJ**
Μean Radius (μm)	7.76	7.35	7.22	6.90	6.43	6.18	6.01	5.69
Standard Deviation	0.48	0.34	0.30	0.33	0.31	0.30	0.29	0.16
Mean monofilament area (μm^2^)	188.9	169.8	163.6	149.8	129.8	120.3	113.5	101.6
Standard Deviation of Monofilament Area	0.71	15.94	0.29	14.28	0.31	11.72	0.26	5.71

## Appendix A

**Table A1:** Raman Data Tables.

**ytd15102117_210**				
**Area**	Center	Width	Shape	I_D_/I_G_
**1.714 × 10^6^**	1293.353	76.866	Gaussian	1.14
**2.322 × 10^6^**	1354.051	36.573	Gaussian	
**9.812 × 10^5^**	1374.569	14.640	Gaussian	
**1.643 × 10^6^**	1523.361	106.078	Gaussian	
**2.750 × 10^6^**	1589.261	40.364	Gaussian	
**ytd15102117_220**				
**Area**	Center	Width	Shape	I_D_/I_G_
**2.184 × 10^6^**	1293.353	87.784	Gaussian	1.04
**2.374 × 10^6^**	1354.051	39.445	Gaussian	
**9.444 × 10^5^**	1374.569	14.738	Gaussian	
**3.052 × 10^6^**	1523.361	106.417	Gaussian	
**2.227 × 10^6^**	1589.261	37.194	Gaussian	
**ytd15102117_230**				
**Area**	Center	Width	Shape	I_D_/I_G_
**2.561 × 10^6^**	1293.353	99.535	Gaussian	1.35
**2.896 × 10^6^**	1354.051	43.540	Gaussian	
**9.155 × 10^5^**	1374.569	14.965	Gaussian	
**2.656 × 10^6^**	1523.361	79.408	Gaussian	
**2.062 × 10^6^**	1589.261	37.930	Gaussian	
**ytd15102117_240**				
**Area**	Center	Width	Shape	I_D_/I_G_
**2.648 × 10^6^**	1293.353	92.829	Gaussian	1.15
**2.662 × 10^6^**	1354.051	44.313	Gaussian	
**9.778 × 10^5^**	1374.569	16.475	Gaussian	
**3.672 × 10^6^**	1523.361	88.085	Gaussian	
**1.772 × 10^6^**	1589.261	37.850	Gaussian	
**ytd15102117_250**				
**Area**	Center	Width	Shape	I_D_/I_G_
**3.537 × 10^6^**	1293.353	98.379	Gaussian	1.21
**2.593 × 10^6^**	1354.051	44.092	Gaussian	
**9.361 × 10^5^**	1374.569	15.943	Gaussian	
**3.965 × 10^6^**	1523.361	88.573	Gaussian	
**1.866 × 10^6^**	1589.261	39.126	Gaussian	
**ytd15102117_260**				
**Area**	Center	Width	Shape	I_D_/I_G_
**3.034 × 10^6^**	1293.353	83.041	Gaussian	0.86
**2.503 × 10^6^**	1354.051	45.529	Gaussian	
**1.018 × 10^6^**	1374.569	18.630	Gaussian	
**5.655 × 10^6^**	1523.361	93.225	Gaussian	
**1.961 × 10^6^**	1589.261	39.163	Gaussian	
**ytd15102117_270**				
**Area**	Center	Width	Shape	I_D_/I_G_
**3.650 × 10^6^**	1293.354	97.955	Gaussian	1.00
**2.638 × 10^6^**	1354.051	45.256	Gaussian	
**8.770 × 10^5^**	1374.569	16.601	Gaussian	
**5.119 × 10^6^**	1523.361	96.119	Gaussian	
**2.049 × 10^6^**	1589.261	40.142	Gaussian	
